# Resource utilization, costs and treatment patterns of switching and discontinuing treatment of MS patients with high relapse activity

**DOI:** 10.1186/1472-6963-13-131

**Published:** 2013-04-08

**Authors:** Karina Raimundo, Haijun Tian, Huanxue Zhou, Xin Zhang, Kristijan H Kahler, Neetu Agashivala, Edward Kim

**Affiliations:** 1 Novartis Pharmaceuticals Corporation, One Health Plaza, East Hanover, NJ 07936-1080, USA; 2 Pharmacotherapy Outcomes Research Center (PORC), University of Utah, Salt Lake City, Utah 84112, USA; 3 KMK Consulting Inc, 215 Ridgedale Avenue, Florham Park, NJ 07932, USA; 4 GCR Medical Affairs, Beijing Novartis Pharma Co. Ltd, Pu Ruan Building, No. 2 Boyun Road, Zhangjiang Hi-Tech Park, Shanghai 201203, China

## Abstract

**Background:**

Multiple sclerosis (MS) is a chronic disease that affects mainly adults in the prime of their lives. However, few studies report the impact of high annual relapse rates on outcomes. The purpose of this study was to identify high relapse activity (HRA) in patients with MS, comparing differences in outcomes between patients with and without HRA.

**Methods:**

A retrospective longitudinal study was conducted using the MarketScan® Commercial Claims and Encounters and Medicare Supplemental Database. Patients had to have at least one ICD-9 for MS (340.XX) in 2009 and one in 2008, be older than 18 years, and have continuous enrolment in the years 2009–2010. HRA was defined as having ≥2 relapses in 2009. Multivariate analyses compared all-cause and MS-specific emergency room (ER) visits, hospitalizations, and all-cause costs, excluding disease modifying therapy (DMT) costs, in 2010 between patients with and without HRA, controlling for baseline characteristics. A subgroup analysis using treatment exposure was also performed.

**Results:**

19,219 patients were included: 5.3% (n=1,017) had ≥2 relapses in 2009. Patients with HRA were more likely to have all-cause and MS-specific resource utilization than patients without HRA. Mean total all-cause non DMT costs were $12,057 higher for the HRA group. In the subgroup analysis, HRA treatment-naïve patients were more likely to start treatment, and HRA treatment-experienced patients were more likely to discontinue or switch index DMT (*P*<0.01).

**Conclusions:**

Patients with ≥2 relapses annually have higher resource utilization and costs. The difference in cost was over twice as large in treatment-naïve patients versus treatment-experienced patients. HRA was also associated with an increased likelihood of starting DMT treatment (treatment-naïve patients), and switching or discontinuing DMT therapy (treatment-experienced patients).

## Background

Relapsing remitting multiple sclerosis (RRMS) is characterized by relapses interspersed between periods of remission. Relapses can last for days, weeks, or months, and cause significant disability and distress [1,2]. Symptoms of multiple sclerosis (MS) include fatigue, spasticity, sensory disturbances, pain, ataxia, tremor, bladder and bowel issues, cognitive effects, weakness, and depression, which affect daily functioning and impact quality of life [2,3]. Furthermore, a strong correlation has been reported between the frequency of relapses and long-term disability, with increased number of relapses early in the course of disease associated with a greater risk of expanded disability status scale (EDSS) worsening over time [4].

Given the clinical presentation of MS, the disease can result in a substantial economic burden. Direct and indirect costs associated with MS in the United States (US) have been estimated at approximately $2.5 billion [5], and because the prevalence of MS appears to be rising, these costs are anticipated also to increase [6]. The total average direct cost of treatment was found to be $47,215 per patient per year (2004 US dollars) [7]. Because disease presentation can vary widely among patients, costs can be significantly different depending on disease severity and frequency of relapses requiring intervention [5]. Treatment costs rise with increasingly complex treatments for individual relapses; treatment complexity is often related to the severity of the relapse. For patients with relapses requiring minimal management (physician visits and symptom-related medication only), the total cost was $243 per episode (2002 US dollars); for relapses requiring moderate intervention (acute hospital, qualifying corticosteroid medication, outpatient follow-up and physical, occupational or speech therapy), the total cost was $1,847 per episode; however, for those requiring a high level of management (acute hospital, post-discharge, rehabilitation facility, skilled nursing facility, short-term nursing home, home healthcare services, and outpatient care), costs rose to $12,870 per episode [5].

Early studies have established a general frequency of relapses of 0.1 to 1.2 per year [8]. We hypothesized that patients with higher frequencies of relapses in a given year would be significantly more costly and use more resources in the following year. The rationale for this hypothesis is that relapse activity is mediated by central nervous system (CNS) inflammatory activity [9]. Therefore, high relapse activity (HRA) may indicate a more refractory or complex form of MS that requires more involved and costly interventions. The objective of this study was to identify and compare resource utilization and costs between patients with and without HRA. In addition, this study aimed to investigate the differences in treatment patterns and costs between patients with and without HRA, stratified by experience with disease modifying therapies (DMTs).

## Methods

### Study design

This was a retrospective, cohort study using MarketScan® Commercial Claims and Encounters Database (CCAE) and the Medicare Supplemental and Coordination of Benefits Database (Medicare Supplement) [10]. The CCAE database represents the healthcare experience of enrolees in commercial health insurance plans sponsored by >100 large-sized and medium-sized employers in the US, which includes monthly enrolment data, inpatient and outpatient medical claims, outpatient prescription drug claims, and eligibility information. The Medicare Supplement includes inpatient and outpatient Medicare supplemental medical claims, linked to drug, person-level enrolment, and benefit plan design data for retirees covered by their previous employers. Both the employer-paid and Medicare-paid components of care are represented in the database. These databases include the health services of employees, dependents, and retirees in the US with primary or Medicare supplemental coverage through privately insured fee-for-service (FFS), point-of-service (POS), or capitated health plans, and are generally representative of the population in the US in terms of gender and age. Because all study data were accessed using techniques compliant with the Health Insurance Portability and Accountability Act, of 1996, informed consent or institutional review board approval was not sought. Novartis Pharmaceuticals Corporation licenses MarketScan Commercial and Medicare databases, and therefore has the right to analyze, interpret, and publish the findings obtained from the dataset.

### Patient populations

#### Main analysis

**Figure 1:**
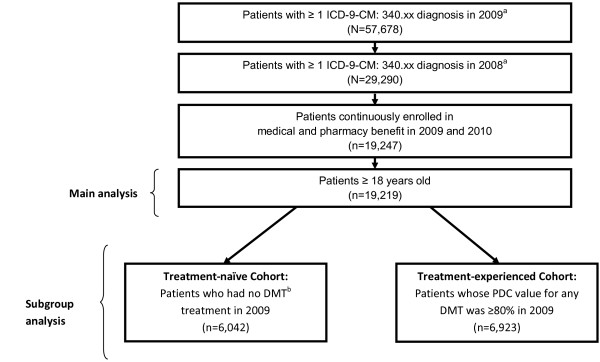
**Patient selection flowchart.** ICD=International Classification of Diseases; CM=Clinical Modification; DMT=disease modifying therapy; PDC=proportion of days covered. ^a^Non rule out diagnosis was used to define patients. ^b^Interferon β-1a, interferon β-1b, glatiramer acetate, or natalizumab.

Patients had to have at least one International Classification of Diseases (ICD)-9-Clinical Modification (CM) for MS (340.XX) in 2009 and one in the prior year, had continuous enrolment in the year of 2009 and 2010, and be 18 years or older in 2009 (Figure 1). Patients were divided into two cohorts: patients with HRA (HRA cohort) who had two or more relapses during 2009, and patients without HRA (non-HRA cohort) who had 0 or 1 relapse during 2009. Relapse was defined according to a validated claims-based relapse detection algorithm [11,12]. The algorithm defined relapses as either: 1) a claim with an MS diagnosis code in the primary position at any time during an inpatient hospitalization, or 2) a claim with an MS diagnosis code in the primary or secondary position in an outpatient setting (including ER visits) in addition to a pharmacy or medical claim for a qualifying corticosteroid on the day of or within 7 days after the visit. A “clean period” of 30 days with none of the events described above was required between the start of separate relapses; otherwise, such claims were incorporated into a single-relapse event.

#### Subgroup definition – treatment-naïve and treatment-experienced

In addition, we performed a subgroup analysis of treatment-naïve and treatment-experienced patients. Patients with no DMT treatment in 2009 were classified as treatment-naïve, while those whose proportion of days covered (PDC) value for any DMT was ≥80% in 2009 were considered treatment-experienced. PDC was calculated as the number of days with drug available divided by the number of days in 2009. The 80% PDC threshold was chosen to ensure that discontinuation or switching treatment, which requires a 60-day gap, would occur in the follow-up period (2010). A PDC ≥80% is generally an adequate threshold to be considered adherent, and thereby to determine the effects of variables on treatment decisions [13]. Few patients using ABCR (Avonex, Betaseron, Copaxone, and Rebif) were billed through J-codes (in-office administration of injectable therapies), and were excluded from the treatment-experienced cohort since these codes are linked to generic names and only indicate standard dosages, thereby not allowing us to differentiate between different DMT dosages or administration mode or identify treatment switches. The majority of the injectable therapies in this study are dispensed through pharmacies which were captured using National Drug Code (NDC) claims. Therapies administered in office, and captured using J-codes, such as natalizumab, were also included in the analysis.

### Statistical analysis

#### Main analysis

Bivariate descriptive statistics compared baseline characteristics of the HRA and non-HRA cohorts with respect to demographics (age, sex, and geographic location), comorbidities, resource utilization, and costs (excluding DMTs) using *t*-tests for continuous variables, and Chi-square tests for categorical variables. Multivariate analyses were performed using longitudinal logistic regression models to compare all-cause and MS-specific ER visits and hospitalizations, controlling for age, gender, geographic region, health plan type, employment status, Charlson comorbidity index (CCI), presence of MS symptoms [3], all-cause ER visits and all-cause hospitalization. All-cause non-DMT costs in 2010 between patients with HRA and those without HRA were compared using a gamma regression with log link model, controlling for the same variables mentioned above. The adjusted costs were predicted for patients with and without and HRA and the cost differences were calculated accordingly. The standard errors and 95% confidence intervals (CI) were provided using paired bootstrapping technique with bias-corrected method [14].

#### Subgroup analysis

Multivariate analysis was performed to compare patients with HRA and those without HRA for the likelihood of starting treatment (naïve cohort), or switching and discontinuing treatment (treatment-experienced cohort) using longitudinal logistic regression models, controlling for age, gender, geographic region, health plan type, employment status, CCI, presence of MS symptoms [3], all-cause ER visits and all-cause hospitalization. Comparisons of all-cause costs (non-DMT costs) were also performed using a gamma regression with log link model in 2010 between patients with HRA and those without HRA, controlling for the same variables mentioned above. Sensitivity analysis was performed for the treatment-experienced cohort to include all patients treated with DMT at some point in 2009, regardless of their PDC to test the impact of the treatment-experience cohort selection criteria on the results. Given that there were no time constraints to the definition of exposure, patients could have had the exposure to DMT and outcome (start, switch or discontinue index DMT treatment) within the same year; thus, a flexible follow-up period censored on Dec 31, 2010 was used.

## Results

A total of 19,219 patients met the study criteria; 94.7% (n=18,202) had less than two relapses and 5.3% (n=1,017) had two or more relapses in 2009 (Table 1). Patients with HRA and those without HRA differed in several characteristics. The HRA cohort was younger (mean age 49.5 vs. 51.6 years) and less likely to be employed (50.2% vs. 56.5%) than the non-HRA cohort (*P*<0.0001 for both). The mean CCI was 0.8 for patients with HRA versus 0.6 for patients without HRA (*P*<0.0001). Patients with HRA reported a higher number of MS symptoms and DMT use in 2009. Patients with HRA had more all-cause ER visits and more all-cause hospitalizations in 2009 (*P*<0.0001 for all).

**Table 1:** Bivariate analyses for baseline characteristics (year 2009)

**Variable**	**Non-HRA cohort**^**a **^**(n=18,202)**	**HRA cohort**^**b **^**(n=1,017)**	***P *value**^**c**^
**Age Group, mean (SD)**	51.6 (11.1)	49.5 (10.6)	**<0.0001**
**Age Group, n (%)**			**<0.0001**
18-35	1,503 (8.3)	107 (10.5)	
36-45	3,671 (20.2)	242 (23.8)	
46-55	6,139 (33.7)	348 (34.2)	
56-65	5,331 (29.3)	274 (26.9)	
65+	1,558 (8.6)	46 (4.5)	
**Gender, n (%)**			0.8502
Male	4,163 (22.9)	230 (22.6)	
Female	14,039 (77.1)	787 (77.4)	
**Geographic Region, n (%)**			**<0.0001**
North East	2,294 (12.6)	143 (14.1)	
North Central	5,926 (32.6)	278 (27.3)	
South	6,137 (33.7)	411 (40.4)	
West	3,830 (21.0)	184 (18.1)	
Unknown	15 (0.1)	1 (0.1)	
**Employment Status, n (%)**			**<0.0001**
Employees	10,279 (56.5)	510 (50.2)	
Spouse/Child/Other	7,923 (43.5)	507 (49.9)	
**Insurance Type, n (%)**			0.8176
Fee for Service (FFS)	14,800 (81.3)	835 (82.1)	
Non-FFS	2,976 (16.4)	159 (15.6)	
Missing	426 (2.3)	23 (2.3)	
**CCI, mean (SD)**	0.6 (1.1)	0.8 (1.3)	**<0.0001**
**Any MS Symptoms, n (%)**^**d**^			**<0.0001**
Yes	12,513 (68.8)	835 (82.1)	
No	5,689 (31.3)	182 (17.9)	
**Any DMT Use, n (%)**			**<0.0001**
Yes	12,408 (68.2)	769 (75.6)	
No	5,794 (31.8)	248 (24.4)	
**All-cause ER Visits**			**<0.0001**
Yes	4,116 (22.6)	386 (38.0)	
No	14,086 (77.4)	631 (62.1)	
**All-cause Hospitalizations**			**<0.0001**
Yes	2,127 (11.7)	238 (23.4)	
No	16,075 (88.3)	779 (76.6)	

MS=multiple sclerosis; SD=standard deviation; FFS=fee for service; CCI=Charlson Comorbidity Index; DMT=disease modifying therapy; ER=emergency room.

^a^Non-HRA cohort: MS relapse equal to 0 or 1 in year 2009.

^b^HRA cohort: MS relapse ≥2 in year 2009.

^c^*P* values of overall comparison: Chi-square for categorical variables; *t*-test for continuous variables.

^d^Including the following list from the National Multiple Sclerosis Society: fatigue, numbness, walking (gait), balance, & coordination problems, bladder dysfunction, bowel dysfunction, visual symptoms, sexual dysfunction, dizziness and vertigo, pain, muscle weakness/spasm/spasticity, cognitive function, depression and other emotional changes, speech disorders, swallowing problems, headache, hearing loss, seizures, tremors, respiration/breathing problems, and itching.

After adjusting for baseline characteristics, patients in the HRA cohort were more likely to have all-cause and MS-specific ER visits and hospitalizations in the following year (Table 2). The presence of MS symptoms and all-cause ER visits or hospitalizations in the prior-period were associated with an increased likelihood of ER visit or hospitalization during the follow-up period.

**Table 2:** Adjusted all-cause or MS-specific ER and hospitalization visits

**Variable**^**a**^	**All-cause ER visits**	**All-cause hospitalizations**	**MS-specific ER visits**	**MS-specific hospitalizations**
**MS Relapse ≥2**	1.33 (1.15-1.54)^b^	1.97 (1.67-2.32)^b^	1.72 (1.43-2.08)^b^	3.25 (2.47-4.29)^b^
**Age group**				
(36–45, Ref: 18–35)	0.81 (0.70-0.93)^c^	0.66 (0.54-0.80)^b^	0.82 (0.67-1.01)	1.20 (0.79-1.83)
(46–55, Ref: 18–35)	0.75 (0.65-0.85)^b^	0.79 (0.66-0.94)^c^	0.70 (0.58-0.85)^d^	0.95 (0.63-1.43)
(56–65, Ref: 18–35)	0.83 (0.72-0.95)^c^	1.02 (0.86-1.22)	0.70 (0.58-0.85)^d^	0.82 (0.54-1.25)
(65+, Ref: 18–35)	1.12 (0.95-1.32)	1.70 (1.38-2.08)^b^	0.71 (0.55-0.90)^c^	0.92 (0.55-1.52)
**Female (Ref: male)**	1.10 (1.01-1.20)^c^	0.95 (0.85-1.06)	1.07 (0.93-1.22)	0.87 (0.68-1.12)
**Region**				
North Central (Ref: Northeast)	1.16 (1.03-1.30)^c^	1.09 (0.94-1.27)	1.60 (1.32-1.95)^b^	1.24 (0.88-1.76)
South (Ref: Northeast)	1.03 (0.91-1.16)	1.01 (0.87-1.18)	1.33 (1.09-1.62)^c^	0.97 (0.68-1.38)
West (Ref: Northeast)	0.95 (0.84-1.08)	0.93 (0.78-1.10)	1.64 (1.33-2.02)^b^	0.82 (0.54-1.22)
Unknown (Ref: Northeast)	1.63 (0.54-4.92)	1.04 (0.22-4.85)	1.08 (0.14-8.55)	7.77 (1.57-38.53)^c^
**Non-Employee (Ref: Employee)**	1.07 (1.00-1.15)	1.06 (0.97-1.17)	1.12 (1.00-1.25)^c^	1.35 (1.09-1.67)^c^
**Plan type**				
HMO and POS capitation (Ref: FFS)	0.94 (0.85-1.04)	0.93 (0.81-1.06)	1.13 (0.98-1.30)	0.80 (0.58-1.11)
Missing (Ref: FFS)	0.76 (0.59-0.98)	0.79 (0.57-1.11)	0.91 (0.63-1.32)	0.67 (0.27-1.65)
**CCI**	1.13 (1.10-1.16)^b^	1.21 (1.17-1.25)^b^	1.04 (1.0-1.09)	1.09 (1.01-1.17)^c^
**Any MS symptoms**	1.53 (1.40-1.67)^b^	1.47 (1.30-1.66)^b^	1.56 (1.36-1.80)^b^	1.70 (1.24-2.33)^c^
**Any DMT use**	0.87 (0.80-0.94)^c^	0.79 (0.72-0.87)^b^	0.96 (0.85-1.07)	1.24 (0.98-1.57)
**All-cause ER visits**	2.50 (2.31-2.71)^b^	1.75 (1.58-1.94)^b^	2.33 (2.07-2.62)^b^	1.86 (1.47-2.35)^b^
**All-cause hospitalizations**	1.37 (1.24-1.52)^b^	2.43 (2.16-2.73)^b^	1.46 (1.27-1.69)^b^	3.12 (2.43-4.01)^b^

*MS*=multiple sclerosis; *Ref*=reference; *HMO*=health maintenance organization; POS=point of service; *FFS*=fee for service; *CCI*=Charlson Comorbidity Index; *DMTs*=disease modifying therapy; ER=emergency room.

^a^Variables were from 2009 and outcomes measured in 2010.

^b^All tests were statistically significant at *P*<0.0001, ^c^*P*<0.05; and ^d^*P*<0.001.

### Costs

Table 3 shows the adjusted mean total annual all-cause costs (excluding DMT costs) for patients with HRA in the follow-up year was $26,803 (95% CI: $24,479-$28,794) compared with $14,745 (95% CI: $14,365-$15,141) for patients without HRA group for a cost difference of $12,057 (95% CI: $9,717-$14,074).

**Table 3:** **Adjusted mean total all-cause costs (excluding DMT costs) in year 2010**^**a**^

	**Mean**	**Standard error**	**Upper level of 95% CI**	**Lower level of 95% CI**
HRA group	$26,803.19	$1126.41	$24,478.92	$28,793.61
Non-HRA group	$14,745.85	$211.09	$14,365.36	$15,140.86
Cost difference	12,057.34	$1149.85	$9,716.94	$14,073.66

^a^Bootstrapping: repeat 200; bias correction method.

*HRA*=high relapse activity; *CI*=confidence interval.

### Subgroup analysis

**Figure 2:**
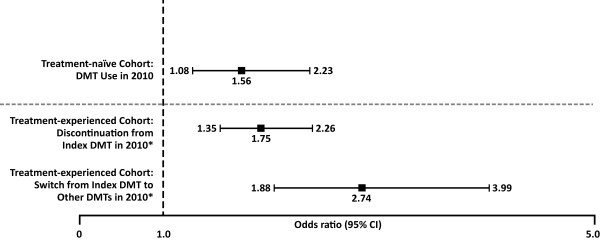
**Likelihood of starting, switching or discontinuing DMT treatment for patients with HRA.** DMT=disease modifying therapy. *The index DMT treatment refers to the last DMT treatment in the year 2009.

The subgroup analysis identified 6,042 patients who were DMT naïve and 13,177 who had prior DMT exposure. Among the prior DMT users, 12,954 patients were eligible for the study (excluding patients with J-codes) and had exposure to DMT in the year 2009; of which, 6,923 (53%) patients had PDC ≥80% and were classified as treatment-experienced (Figure 1). Among treatment-naïve patients, those with HRA were more likely to start DMT use than those without HRA in the follow-up period [odd ratio (OR) 1.56, 95% CI: 1.08-2.23; Figure 2]. Among treatment-experienced patients (PDC ≥80%), HRA was associated with a greater likelihood of discontinuing the index DMT (OR=1.75, 95% CI: 1.35-2.26) or switching from the index DMT to another DMT (OR=2.74, 95% CI: 1.89-3.99) than patients without HRA in the follow-up period (Figure 2).

Excluding DMT-related costs, total healthcare costs for 2010 for this subgroup analysis of treatment-naïve and treatment-experienced populations were similar to the overall group in that patients with HRA had higher costs than those without HRA. In the treatment-naïve cohort, costs were $33,855.63 (95% CI: $28,050.17-$40,945.48) in those with HRA versus $18,100.61 (95% CI: $17,260.92-$18,994.27), for a cost difference of $15,755.03 (95% CI: $9,321.56-$22,770.56; data not shown). In the treatment-experienced cohort, costs were $19,849.38 (95% CI: $17,624.66-$22,317.70) in those with HRA and $12,243.92 (95% CI: $11,767.29-$12,798.37) in those without HRA, for a cost difference of $7,605.45 (95% CI: $5,281.50-$10,096.58; data not shown).

### Sensitivity analysis

The sensitivity analysis included all patients with exposure to DMT in 2009, regardless of their PDC, in the treatment-experienced cohort. We identified 12,954 eligible patients, of which 748 (5.8%) had HRA. Patients with HRA were more likely to discontinue their DMT before Dec 31, 2010 (OR: 1.47, 95% CI: 1.26-1.71), or switch from the index DMT to another DMT before Dec 31, 2010 (OR=2.41, 95% CI: 1.90-3.06) than patients without HRA (data not shown).

Excluding DMT-related costs, total differences in healthcare costs for 2010 for the experienced subgroup sensitivity analysis was larger than the treatment-experienced cohort in the main analysis. Total costs were $23,573.50 (95% CI: $21,579.51- $25,799.29) in those with HRA and $13,140.34 (95% CI: $12,600.33-$13,546.19) in those without HRA, for a cost difference of $10,433.16 (95% CI: $8,545.54-$12,735.78; data not shown).

## Discussion

### Main analysis

This retrospective claims database study showed that patients with MS and HRA have greater resource utilization and costs than patients without HRA. The likelihood of all-cause and MS-specific ER visits and hospitalizations was significantly greater for patients with HRA than for those without HRA. Because relapse frequency changes slowly over time, all-cause and MS-specific ER visits and hospitalizations in 2009 increased the likelihood of similar visits in 2010. Patients with HRA were younger than those with fewer relapses, consistent with previous reports that have shown that relapse frequency diminishes over time [15]. The direct annual non-DMT cost of patients with MS and HRA was $12,057 greater than patients without HRA. These results were expected given that more frequent relapses likely indicate a more complex, refractory disease course that may require more involved management, including costly hospitalizations. In this regard, HRA may reflect an underlying pathology rather than a state of MS characterized by ongoing CNS inflammation despite clinical management. A previous retrospective study of a managed care medical and pharmacy claims database found that patients with no relapses cost an annual average of $6007 (1995–96 US dollars) while those who experienced two relapses had total costs of $14,521, and those with more than two relapses had costs totaling $20,519 [16].

### Subgroup analysis

Disease modifying treatments are important tools for the management of MS, reducing relapse frequency and accumulation of irreversible disability. However, patients may have suboptimal responses to first-line therapy, prompting a switch to a different DMT or discontinuation of all therapy [17]. Our subgroup analysis found that treatment-naïve patients with HRA were more likely to start DMT treatment, while treatment-experienced patients with HRA were more likely to switch or discontinue DMT therapy. Our sensitivity analysis showed that using a less conservative definition of treatment-experience, where all patients with DMT use in year 2009 were included in the analysis regardless of their PDC, yielded similar results. The small reduction in the sensitivity analysis is explained by the fact that patients could have started and switched or discontinued their DMT treatment in the same year (2009), while in the main treatment-experienced cohort, we were able to identify patients who switched or discontinued in the follow-up year (2010) only. The latter is a more precise approach since we are able to separate exposure to the outcome that was being predicted.

The results of the subanalysis are compatible with the work of Visser et al. (2011) [18] that found that 66% of patients who switched DMTs cited progression of disease as the reason for the switch, while 53% of those who discontinued DMT treatment indicated that uncertainty about efficacy had a “very big influence” or a “big influence” on why they discontinued DMT [18]. What is striking in our study is that, despite a higher probability of treatment switching, HRA patients continued to have high costs in the subsequent year. This suggests that switching among existing DMTs may not be effective in managing relapse activity. A study by Prosperini et al. (2012) [19] indicated that switching among self-injectable DMTs was not associated with benefits, but escalation to a different mechanism of action with higher potency did increase the duration of disease-free activity [19]. Additional studies with more recent data are needed to examine the differential effect of high-efficacy options such as fingolimod and natalizumab on reducing costs among patients with HRA.

### Limitations

There are a few limitations to be acknowledged in this study. The first limitation of this analysis is that there is no known HRA definition; however, the authors believed that having 2 or more relapses a year is more than the estimated yearly rates of 0.2 to 1.2 [8]. In addition, the definitions of MS relapse and thresholds for intervention may vary among clinicians, and the algorithm used to identify relapses was based on the treatments received. Therefore, the true number of relapses may be underestimated. The present investigation into costs associated with relapses did not include DMTs because their use is not restricted to the time of relapse and are instead, used on a continued basis for long-term management. The costs of DMTs contribute substantially to the total costs of managing MS, and the objective of the current study was to assess medical costs, which are a proxy for ongoing disease activity, that generate non-drug costs, as well as lowered quality of life and increased risk for accumulation of irreversible disability; however, this was out of the scope of this work. Finally, resource utilization and costs were evaluated over a one-year follow-up period; a longer time horizon may be necessary to assess long-term outcomes and costs.

## Conclusions

This analysis shows that patients with two or more relapses in a given year have higher resource utilization and are more costly in the following year than those with less than two relapses. HRA predicts DMT initiation among treatment-naïve patients, and switching or discontinuation DMT among treatment-experienced patients. Future research should investigate the effects of relapse activity on long-term costs.

## Competing interests

KR was a fellow of Novartis Pharmaceuticals Corporation at the time the research was conducted and at manuscript submission. HT, HZ, KHK, NA, and EK are employees of Novartis Pharmaceuticals Corporation. XZ is a KMK Consulting consultant who is currently working for Novartis Pharmaceuticals Corporation. Funding for the development of this manuscript was supplied by Novartis Pharmaceuticals Corporation. Write All, Inc. received funding for its services from Novartis Pharmaceuticals Corporation.

## Authors' contributions

KR, HT, HZ, XZ, KHK, NA, and EK were involved in the initiation of the study and in the definition of study objective and search strategy. Each of the authors also aided in conducting the study analysis and interpreting the study results, and wrote and critically reviewed the manuscript. All authors have read and approved the entire manuscript.

## Pre-publication history

The pre-publication history for this paper can be accessed here:

http://www.biomedcentral.com/1472-6963/13/131/prepub
